# Is There a Potential Therapeutic Role for Caveolin-1 in Fibrosis?

**DOI:** 10.3389/fphar.2017.00567

**Published:** 2017-08-24

**Authors:** Waled A. Shihata, Mohammad R. A. Putra, Jaye P. F. Chin-Dusting

**Affiliations:** ^1^Vascular Pharmacology Laboratory, Cardiovascular Disease Program, Department of Pharmacology, Biomedical Discovery Institute, Monash University Clayton, VIC, Australia; ^2^Department of Medicine, Monash University Clayton, VIC, Australia; ^3^Baker Heart and Diabetes Institute Melbourne, VIC, Australia

**Keywords:** caveolae, caveolin-1, cardiac fibrosis, lung fibrosis, kidney fibrosis

## Abstract

Fibrosis is a process of dysfunctional wound repair, described by a failure of tissue regeneration and excessive deposition of extracellular matrix, resulting in tissue scarring and subsequent organ deterioration. There are a broad range of stimuli that may trigger, and exacerbate the process of fibrosis, which can contribute to the growing rates of morbidity and mortality. Whilst the process of fibrosis is widely described and understood, there are no current standard treatments that can reduce or reverse the process effectively, likely due to the continuing knowledge gaps surrounding the cellular mechanisms involved. Several cellular targets have been implicated in the regulation of the fibrotic process including membrane domains, ion channels and more recently mechanosensors, specifically caveolae, particularly since these latter contain various signaling components, such as members of the TGFβ and MAPK/ERK signaling pathways, all of which are key players in the process of fibrosis. This review explores the anti-fibrotic influences of the caveola, and in particular the key underpinning protein, caveolin-1, and its potential as a novel therapeutic target.

## Introduction

Fibrosis, a process of wound reparation, is a cellular response to tissue damage or insult, preceded by an inflammatory response. Various etiologies can act as triggers to induce inflammation and subsequent tissue damage, including infection, autoimmunity and tumors (Wynn, 2007; Wick et al., 2013). In physiological conditions, inflammation is followed by repair processes that eliminate the initial insult, through the replacement and replenishment of injured cells. The repair process progresses with the replacement of connective tissue or fibrous tissue-producing mesenchymal cells such as fibroblasts, the primary cells involved in fibrosis. Of note, this resolution step initiates the recruitment of leukocytes and subsequently promotes the process of apoptosis (Akbar and Salmon, 1997; Buckley et al., 2001). When the resolution step becomes dysregulated, observed by a transition from an acute to a chronic inflammatory process, fibrosis occurs which manifests as scar tissue, further promoting end-organ damage and ultimately death (Wynn, 2007; Wick et al., 2013). Chronic inflammation is essential for the progression of fibrosis as it promotes long-term tissue remodeling and subsequent organ dysfunction (Wynn, 2007; Wick et al., 2013; Zeisberg and Kalluri, 2013). The onset of fibrosis begins with epithelial or endothelial activation, which results in the release of inflammatory mediators, for both clot formation (hemostasis) and inflammatory cell recruitment. Innate immune cells, such as neutrophils, release pro-fibrotic factors or cytokines, including matrix metalloproteinases (MMPs), elastases, and cathepsins, to cleave connective tissue and in turn disrupt the basement membrane, promoting cell migratory capacities (Wynn, 2007; Wick et al., 2013). In addition, macrophages are vital for fibroblast activation, through their release of tumor necrosis factor-α (TNF-α) and interleukin 1 (IL-1). Further, macrophages also release transforming growth factor β (TGF-β), which is essential for the onset of processes involved in fibrosis including epithelial-mesenchymal transformation (EMT) (Wick et al., 2013). Morphologically, caveolae are described as flask-like or Ω shaped membrane invaginations with a diameter of 50–100 nm (Palade, 1953; Yamada, 1955). There are two main protein components essential for the formation and function of caveolae—caveolins and cavins (Drab et al., 2001; Galbiati et al., 2001). The primary function of caveolae is yet to be determined, although it has been shown to play a mediatory role in cholesterol homeostasis (Frank et al., 2008; Zhang et al., 2008), vascular reactivity (Drab et al., 2001; Razani et al., 2001a), mechanotransduction (Park et al., 1998; Sinha et al., 2011; Shihata et al., 2016), and cellular signaling (Yang and Rizzo, 2007). Importantly, caveolin-1 (Cav-1), originally called vesicular integral membrane protein of 21 kD (VIP-21), is generally accepted as the predominant protein involved in caveolae's key regulatory function in intracellular signaling pathways evident in fibrotic processes observed in different organs including cardiac, lung, and kidney fibrosis (Drab et al., 2001).

## Cardiac fibrosis

Cardiac fibrosis is the abnormal accumulation of extracellular matrix (ECM) in the myocardial tissue. Key to the pathogenesis of cardiac fibrosis is the limited regenerative ability of the adult mammalian heart (Soonpaa and Field, 1998). This is due to the transition phase during neonatal stages, where there is a switch from hyperplastic to hypertrophic growth, observed by an increase of myocardial mass, independent of proliferation. In the event of cardiac damage or injury, an inflammatory process is followed by the replacement of cardiomyocytes with fibrotic tissue in place of a replenished supply of cardiomyocytes (Soonpaa and Field, 1998). In the cardiac setting, Cav are present as various subtypes, however Cav-1 and Cav-3 are the predominant isoforms expressed by endothelial cells and cardiomyocytes, respectively (Tang et al., 1996).

The deletion of the gene encoding Cav-1 leads to adverse cardiac remodeling following myocardial infarction (MI). It has been demonstrated that this is due to Cav-1 knockout (Cav1^−/−^) mice having an impaired inflammatory reaction, where alternative or M2 macrophages, known for promoting scar formation, accumulate more in Cav1^−/−^ mice compared to WT mice. Further adoptive transfer studies, where macrophages from Cav1^−/−^ mice were transferred into WT mice and vice versa, showed an increase in the survival rate of Cav1^−/−^ mice that received macrophages from WT mice and decreased survival rates for WT mice who received Cav1^−/−^ macrophages (Shivshankar et al., 2014). Similarly, a study evaluated the degree of fibrosis in the heart of mice with overexpression of Cav-3 compared to WT mice. Following transverse aortic constriction (TAC) procedures, mice that overexpressed Cav-3 displayed lower levels of fibrosis, and improved natriuretic levels, relative to the WT mice (Horikawa et al., 2011). Further, it has been shown that reconstitution of endothelial Cav-1 into Cav-1^−/−^ knockout mice reduces gene expression of TGF-β1, collagen I and III, ultimately decreasing fibrotic lesions in the heart and improving cardiac function (Murata et al., 2007). Importantly, several studies have highlighted the major contributory role of TGF-β in the pathogenesis and regulation of cardiac fibrosis and hypertrophy (Brooks and Conrad, 2000; Deten et al., 2001; Dewald et al., 2004).

The main regulatory mechanism for TGF-β signal transduction is endocytosis regulated by Cav-1-associated lipid rafts and early endosomal antigen-1 (EEA-1) non-lipid rafts. While EEA-1 increases TGF-β1 signaling, Cav-1-associated internalization reduces, and in some cases even abolishes TGF-β1 signaling (Di Guglielmo et al., 2003; Ito et al., 2004). Without downregulation from Cav-1, ligand-receptor binding of TGF-β1 initiates the assembly of a heteromeric receptor complex by transphosphorylating the TGF-β type II receptor (TβRII), which then activates TGF-β type I receptor (TβRI) (Wrana et al., 1994). Further, TβRI induces Smad signaling by phosphorylating Smads, receptor-regulated Smad (R-Smad) 2 and 3. Smad2 and Smad3 relay the signaling process from the plasma membrane to the nucleus, via R-Smads and Smad4 that translocate to the nucleus where transcriptional changes occur (Miyazono et al., 2000). Cav-1 negatively regulates Smad signaling by binding the caveolin scaffolding domain (CSD) component, the key functional component of Cav-1, to TβRI, which in turn diminishes the downstream signaling of Smads via the TGF-β receptors (Razani et al., 2001b). The interaction between Cav-1 and TβRI impairs the phosphorylation process, and in turn inhibits the heteromerization with Smad4, which is essential for the initiation of transcriptional changes (Razani et al., 2001b). In addition to Smad signaling pathways, TGF-β also exerts its actions through non-canonical, non-Smad pathways including Mitogen-activated protein kinase (MAPK) pathways, Rho-like GTPase signaling pathways, and phosphatidylinositol-3-kinase/Protein Kinase B (PI3K/AKT) pathways. Cav-1 has also been demonstrated to negatively regulate the activation state of p42/44 MAPK cascade in cardiac fibroblasts (Galbiati et al., 1998). This finding is also supported by morphological evidence demonstrating ERK-1/2 co-localization to caveolae in an *in vivo* model of cardiac fibrosis (Liu et al., 1997).

## Lung fibrosis

Lung fibrosis, such as idiopathic pulmonary fibrosis (IPF) and nonspecific interstitial pneumonitis, is the excess accumulation of ECM in lung tissue. IPF is defined as a chronic, progressive lung fibrosis condition without a specific cause and a mild level of inflammation, predominantly in older populations (Raghu et al., 2011; Travis et al., 2013). The role of Cav-1 in lung fibrosis was investigated by Kasper et al. where they induced fibrosis by irradiating alveolar cell types I and II of rats and mini pigs (Kasper et al., 1998). This study revealed downregulated Cav-1 gene expression in epithelial cells following radiation, resulting in fibrosis. Morphologically, the epithelial cells showed reduced expression of caveolae, coupled with attenuated signal transductory capacities (Kasper et al., 1998). Moreover, a five-fold increase in collagen expression via Mitogen-activated protein kinase kinase/extracellular signal-regulated kinase (MEK/ERK) activation was observed in human lung fibroblasts when Cav-1 gene expression was reduced by 70% (Tourkina et al., 2005). Similarly, a study by Wang et al. observed a two-fold decrease in Cav-1 gene expression in lung tissue from IPF patients compared to control patients (Wang et al., 2006b). Furthermore, they investigated the therapeutic effects of Cav-1 in rat lungs using bleomycin, an antineoplastic antibiotic that causes pulmonary fibrosis as a major side effect. In another injury model, mice transfected with an adenovirus vector containing the Cav-1 gene, demonstrated resolved lung fibrosis, observed by reduced fibrotic area in the lung relative to untreated mice (Wang et al., 2006b). Like cardiac fibrosis, the formation of scar tissue in lung fibrosis is also driven by TGF-β (Khalil et al., 1991, 1994, 1996; Verma and Slutsky, 2007; Del Galdo et al., 2008). *In vitro* models of lung fibrosis have demonstrated that the anti-fibrotic effects observed are regulated by TGF-β1 via ERK and JNK activation, which are mediated by Cav-1. Indeed, TGF-β1 induces the gene expression of collagen type I via ERK1, while it regulates the production of fibronectin via c-Jun N-terminal kinase 1 (JNK1) (Wang et al., 2006b). Of note, TGF-β signaling can also mediate Cav-1 expression via non-SMAD signaling pathways. Indeed, downregulated Cav-1 has been observed in TGF-β-treated human lung fibroblasts through the activation of p38 MAPK (MEK/ERK signaling) (Sanders et al., 2015). The anti-fibrotic therapeutic potential of Cav-1 has further been highlighted in studies utilizing pharmacologically administration of CSD peptide. Similar to knockout studies, collagen deposition was reduced by more than 95% in less than 5 h in mice treated with the CSD peptide relative to the untreated mice (Tourkina et al., 2008). CSD peptide-dependent reductions in fibrosis were accompanied by inhibitions in MEK, ERK, JNK, and AKT activity as well as altering their cellular localization, confirming the anti-fibrotic effects of Cav-1 (Tourkina et al., 2008).

Like EMT, Endothelial-mesenchymal transition (EndoMT) has emerged as a key source of fibroblasts and myofibroblasts critical in the pathogenesis of fibrotic disease (Piera-Velazquez et al., 2011; Lin et al., 2012). EndoMT has been shown to be regulated via TGF-β (Li and Jimenez, 2011), with TGF-β being a potent inducer of EndoMT in pulmonary endothelial cells via the transcription factor, SNAIL1. In addition, the internalization of TGF-β receptors expressed on the cell membrane have been attributed to the cellular capabilities of Cav-1 (Razani et al., 2001b). Interestingly, pulmonary endothelial cells isolated from Cav-1^−/−^ mice spontaneously underwent EndoMT. This cellular transition was further increased with the administration of TGF-β, highlighting the importance of both pathways (Li et al., 2013). Although the exact mechanisms involved in EndoMT are relatively unclear, SNAIL1 has been found to be a requirement for the induction of this process (Kokudo et al., 2008). This is supported by increased SNAIL1 gene expression observed in Cav1^−/−^ mouse endothelial cells, which is reversed by the reconstitution of Cav-1, emphasizing the key mediatory role of Cav-1 in EndoMT (Strippoli et al., 2015).

Fibroblast proliferation is normally regulated by polymerized collagen via the inhibition of the PI3K-AKT-S6-kinase 1 (S6K1) signal pathway, by high phosphatase and tensin homolog (PTEN) (Xia et al., 2008, 2010). This negative feedback mechanism limits the proliferation of fibrotic tissue following injury in physiological conditions, however when the process is impaired, fibrotic conditions such as IPF may occur (White et al., 2006). Protein expression of both Cav-1 and PTEN was significantly reduced in the cellular membrane of myofibroblasts within fibroblast foci in lung cells of IPF patients' relative to control patients, compared to surrounding epithelial cells. In addition, a correlation in Cav-1 and PTEN levels was observed, which could be attributed to PTEN suppressing PI3K/AKT activation (Xia et al., 2008, 2010). This occurs through the translocation from the cytoplasm into the cellular membrane, where it is activated and in turn inhibits the signaling process. Cav-1 as an integral protein regulates PI3K/AKT transduction, where augmented expression of Cav-1 subsequently results in reduced levels of PTEN in the cellular membrane. This phenomenon is observed in Cav-1 null mice which demonstrate low expression of PTEN in the cellular membrane compared to wild type mice. Following reconstitution of Cav-1 using an adenovirus vector in the Cav-1 null fibroblast cells, a higher association of PTEN with cellular membrane and lower PI3K/AKT signaling was observed. This association was further supported by amino acid sequence analysis, showing a specialized binding sequence domain for PTEN which directly interacts with Cav-1 (Xia et al., 2008, 2010). Similarly in cardiac fibrosis and IPF, a downregulation in both Cav-1 and PTEN protein expression was observed in cardiac and pulmonary fibroblasts, respectively, relative to controls (Gao et al., 2014).

Further, MAPK pathways have been reported to play a mediatory role in TGF-β1 signaling, key for fibrotic processes, and have also been shown to be regulated by Cav-1 (Yue and Mulder, 2000; Fujita et al., 2004; Wang et al., 2006a). It has been demonstrated that Cav-1 regulates TGF-β1-induced ECM production via MAPK pathway in the lung. Over-expression of Cav-1 via transfection of adenovirus vector inhibited TGF-β1-induced ERK and JNK activation, consequently resulting in decreased ECM production (Wang et al., 2006b). Despite the above findings highlighting the anti-fibrotic role of Cav-1, studies have also demonstrated contrasting roles for Cav-1 in tissue fibrosis. Cav-1 deficiency has been linked with significant inhibition of premature senescence of fibroblasts (Volonte and Galbiati, 2009), whereas elsewhere it has been shown that Cav-1 is needed to induce senescence in fibroblasts (Dasari et al., 2006). Indeed, Cav-1^−/−^ mice were found to be protected against fibrosis induced by bleomycin relative to WT mice (Shivshankar et al., 2012). When exposed to bleomycin to induce fibrosis, WT mice displayed significant collagen deposition, whereas Cav-1^−/−^ mice who did not present a significant increase (Shivshankar et al., 2012). This blunted fibrotic response could be due to reduced epithelial cell senescence and apoptosis in the Cav-1^−/−^ mice. However, in contrast to these findings, a study demonstrated increased fibrosis, upregulated apoptosis and cellular senescence in Cav-1^−/−^ mice exposed to bleomycin (Linge et al., 2007). The disparity in these findings could be due to the timing of fibrotic measurements and the expression of Cav-1, where it is very likely that in the early stages of exposure to bleomycin, Cav-1 is expressed which is linked with upregulated apoptosis and senescence in epithelial cells, whereas in the later stages, Cav-1 is decreased, which may be associated with the progression of fibroblast-mediated fibrosis (Shivshankar et al., 2012).

## Peritoneal and kidney fibrosis

Patients with terminal stage of kidney failure require either hemodialysis or peritoneal dialysis (PD). The common adverse effects following frequent long term PD is acute inflammation, which leads to chronic inflammation and eventually fibrosis (Grassmann et al., 2005; Strippoli et al., 2015). The predominant cause of acute inflammation is the continuous exposure of the peritoneal membrane of the kidney to hyperosmotic, hyperglycaemic, and acidic dialysis solutions. This leads to a specific EMT process of the mesothelial cells, known as mesothelial-to-mesenchymal transition (MMT). Like EMT, MMT is characterized by features such as loss of E-cadherin and cytokeratin, and upregulated α-smooth muscle actin (α-SMA) and fibroblast specific protein-1 (FSP-1) expression (Yanez-Mo et al., 2003; Strippoli et al., 2015). Of note, TGF-β is one of the early pro-fibrotic factors detected during PD treatment in human patients relative to control patients (Yanez-Mo et al., 2003). Further, p38 is also implicated as a key regulator of MMT, slowing down the transition process by promoting E-cadherin gene expression. E-cadherin is important for cell-to-cell adhesion and it is preserved by p38 via its inhibition of TGF-β via TGF-β-activated kinase 1-nuclear factor kappa-light-chain-enhancer of activated B cells (TAK1-NF-κB) signaling (Strippoli et al., 2010). The main suppressor of E-cadherin is the transcription factor SNAIL1, which modulates its effects via ERK or NF-κB. SNAIL1 is known as one of the major inducers of EMT and is also implicated in the downregulation of cytokeratin gene expression (Strippoli et al., 2008). Studies using Cav-1 gene silencing in human primary mesothelial cells (HPMCs) as well as mesothelial cells from Cav-1^−/−^ mice have shown a loss of the typical cell structure and the acquisition of a spindle-like shape, characteristic of fibroblasts. This morphological change is also accompanied by suppression of E-cadherin gene expression. In addition, Cav-1^−/−^ mice presented with increased α-SMA and collagen type I gene expression coupled with hyper-activation of ERK1 and ERK2, even in the basal state, relative to the WT mice (Strippoli et al., 2015). This was attributed to the inhibitory nature of Cav-1 on the ERK1/2 pathway. Moreover, HPMCs deficient of Cav-1 presented with enhanced ERK1/2 activity when stimulated by TGF-β. The hyper-activation of ERK1/2 leads to a stronger repression of E-cadherin gene expression via SNAIL1, accelerating the progression of MMT to fibrosis. Lentiviral overexpression of Cav-1 in HPMCs restored and reduced E-cadherin and α-SMA gene expression, respectively (Strippoli et al., 2015). The role of Cav-1 in MMT could be ascribed to the activation of ERK1/2, which directly upregulates SNAIL1 and suppresses E-cadherin and or to the diminished Smad2/3 signaling through the inhibitory effects of Cav-1 on TGF-β receptors (Xu Y. et al., 2008).

However, similar to fibrotic conditions in the lung, the role of Cav-1 is not definitive in kidney fibrosis, with several studies suggesting that Cav-1 plays a critical role in maintaining the fibrotic condition. A study conducted by Chen et al. (2012) demonstrated increased epidermal growth factor receptor (EGFR) association with phosphorylated Cav-1 in renal proximal tubular epithelial cells exposed to angiotensin II (AngII). An increase in reactive oxygen species (ROS) production was observed following angiotensin type 1 receptor (AT1R) activation, promoting Src kinase activation, Cav-1 and EGFR phosphorylation and consequently resulting in prolonged EGFR-ERK signaling which induces prolonged EMT (Chen et al., 2012). Further, this study also observed that silencing Cav-1 gene via small interfering RNA (siRNA) or via knockdown model leads to inhibited AngII activation, suggesting that EGFR association with Cav-1 leads to prolonged activation (Chen et al., 2012). These findings are in contrast to studies which have concluded that EGFR interactions with Cav-1 in caveolae or lipid rafts result in the inactivation of the EGFR (Orth and McNiven, 2006). Moreover, Chen & colleague's findings contradict a study conducted by Forrester et al. (2017) which found worsened perivascular fibrosis and hypertrophy in kidney tissue of Cav-1^+/+^ compared to Cav-1^−/−^ mice when administered with AngII, coupled with attenuated VCAM-1 expression in the endothelium and adventitia layer of Cav-1^−/−^ mice relative to Cav-1^+/+^ mice (Forrester et al., 2017).

## Liver fibrosis

Liver cirrhosis, the end-stage of various liver disease, is characterized by the replacement of normal physiologic hepatocyte cells with fibrotic tissue and ultimately organ failure (Elsharkawy et al., 2005; Asrani et al., 2013). Liver injury commonly results from “sinusoidal” portal hypertension, primarily caused by intrahepatic shunts and hepatocyte swelling (Sherman et al., 1990; Yokomori et al., 2002). A key factor involved in liver cirrhosis is endothelial nitric oxide synthase (eNOS), which regulates the blood flow, a central parameter in portal hypertension, through its production of the potent vasodilator, nitric oxide (NO). Indeed, with reduced activity of eNOS, the liver is more susceptible to portal hypertension, leading to fibrosis and eventually cirrhosis (Matei et al., 2006). Cav-1 is a major negative regulator of eNOS, which impacts the enzyme through direct interaction of both C- and N- terminals of Cav with oxygenase domain of eNOS (Ju et al., 1997). Unlike in most organs discussed, Cav-1 is suggested to play a pro-fibrotic role in the liver. In fact, Cav-1 protein levels are increased in experimental liver disease as well as a murine model of Niemann-Pick disease type C (NPC), a disease characterized by impaired cholesterol homeostasis (Garver et al., 1997; Shah et al., 1999). Moreover, rats treated with dimethylnitrosamine (DMN) to induce liver cirrhosis demonstrate enhanced Cav-1-eNOS binding paired, with a positive correlation between Cav-1 protein expression and the degree of liver fibrosis (Xu B. et al., 2008). Similarly, Cav-1 was localized in liver sinusoidal endothelial cells (LSECs) of cirrhotic liver human samples, with an overexpression of Cav-1 in late-stage cirrhosis (Yamazaki et al., 2013). The role reversal of Cav-1 in liver fibrosis could be due to the fact that the cirrhotic liver contains higher levels of cholesterol, and Cav-1 is a major cholesterol binding protein, thus Cav-1 expression may be stimulated in chirrotic liver (Bist et al., 1997).

## Conclusion

**Figure 1:**
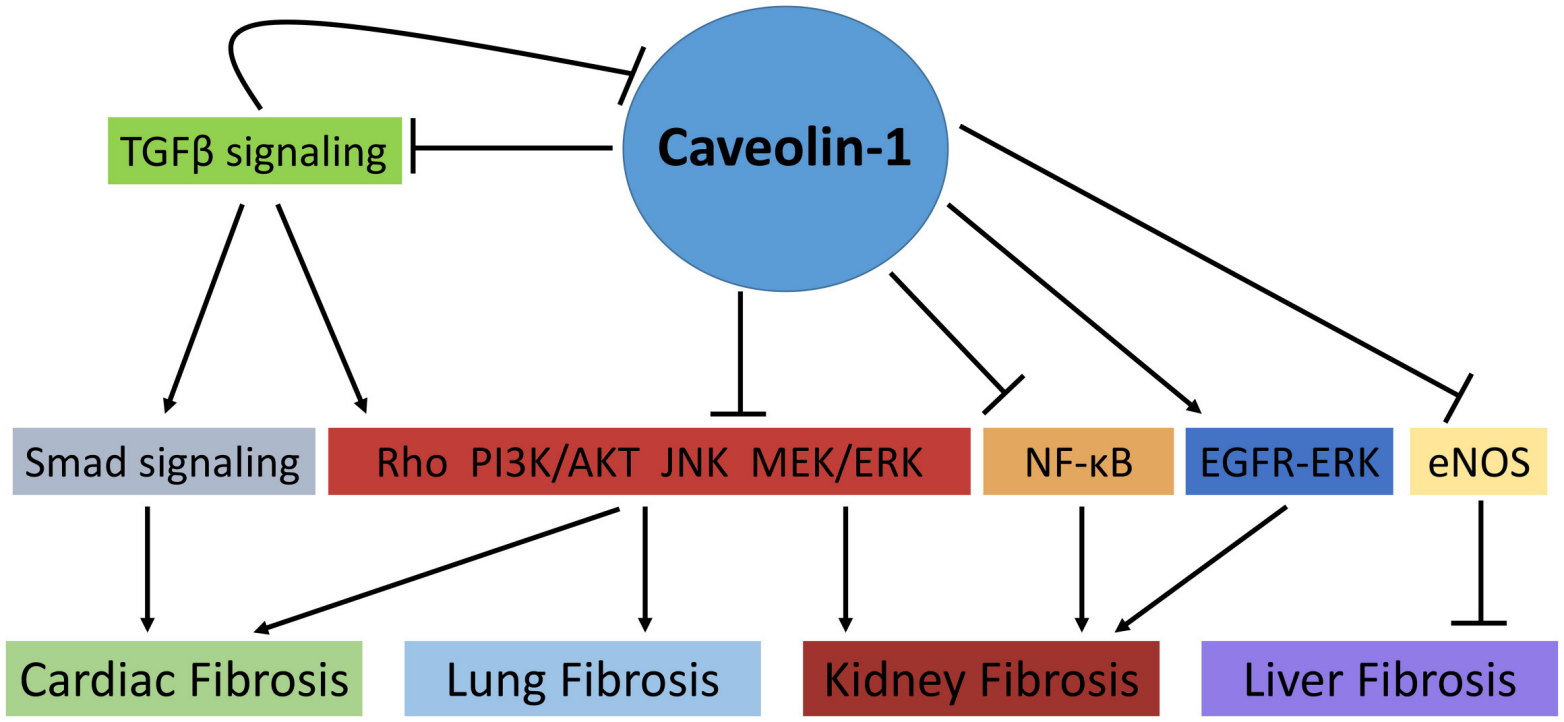
The role of Caveolin-1 in cellular signaling mechanisms involved in fibrosis. Caveolin-1 (Cav-1) directly and indirectly regulates fibrotic processes in various tissues. In cardiac and lung fibrosis, Cav-1 prevents collagen deposition, fibroblast proliferation and TGFβ signaling through its negative regulation of Smad and non-Smad signaling pathways such as Rho-like GTPase, PI3K/AKT, MAPK (MEK/ERK), and JNK signaling pathways. Similarly, in kidney fibrosis, Cav-1 modulates fibrotic processes via the aforementioned pathways as well as NF-κB signaling. Of note, TGFβ signaling can also mediate Cav-1 expression via the activation of non-SMAD signaling pathways. Conversely, Cav-1 has been shown to promote kidney fibrosis by prolonging EGFR-ERK signaling. Moreover, in liver fibrosis, Cav-1 promotes liver cirrhosis through its negative regulation of eNOS (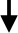, activation; 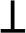, inhibition).

The pathophysiology of organ fibrosis has been studied extensively, and the anti-fibrotic role of caveolae and caveolins has been explored. Indeed, novel studies using both animal and pharmacological models have identified Cav-1 as a potential anti-fibrotic target in a number of fibrotic settings including cardiac, lung, and kidney fibrosis. Although the relationship between Cav-1 and key regulatory molecules involved in fibrotic processes such as TGF-β, ERK1/2, and Smad is understood extensively, it is still relatively unclear how they interact at the intracellular level and whether these Cav-1-associated signaling pathways can be targeted as potential therapies in fibrosis. Moreover, the tissue-specific effects of Cav-1 highlights the gap in knowledge regarding its role in fibrotic conditions. Indeed, contrasting studies highlight the dynamic role of Cav-1 in organ fibrosis, specifically in the kidney and lungs (Figure 1). Thus, further studies are essential to completely understand possible therapeutic effects of caveolae/caveolin-1 in fibrosis and whether its therapeutic potential is limited between varying human fibrotic conditions.

## Author contributions

WS and MP drafted the manuscript and contributed to the interpretation of data in the review. WS prepared the manuscript. WS, MP, and JC reviewed, edited, and approved the final version of the manuscript.

### Conflict of interest statement

The authors declare that the research was conducted in the absence of any commercial or financial relationships that could be construed as a potential conflict of interest.

## Abbreviations

^−/−^: knockout
AKT: Protein Kinase B
AngII: angiotensin II
AT1R: angiotensin type 1 receptor
Cav-: caveolin-
CSD: caveolin scaffolding domain
ECM: extracellular matrix
EEA-1: endosomal antigen-1
EGFR: epidermal growth factor receptor
EMT: epithelial-mesenchymal transformation
EndoMT: Endothelial-mesenchymal transition
ERK: extracellular signal-regulated kinase
FSP-1: fibroblast specific protein-1
HPMCs: human primary mesothelial cells
IL-: Interleukin-
IPF: Idiopathic pulmonary fibrosis
JNK: c-Jun N-terminal kinase
MAPK: Mitogen-activated protein kinase
MEK: Mitogen-activated protein kinase kinase
MI: myocardial infarction
MMP: matrix metalloproteinase
MMT: Mesothelial-mesenchymal transition
NF-κB: nuclear factor kappa-light-chain-enhancer of activated B cells
PD: Peritoneal dialysis
PI3K: phosphatidylinositol-3-kinase
PTEN: phosphatase and tensin homolog
R-Smad: Receptor-regulated Smad
S6K1: S6 kinase 1
siRNA: Small interfering RNA
TAK1: TGF-β-activated kinase 1
TGF-β: transforming growth factor β
TNF-α: tumor necrosis factor-α
TβRI: TGF-β type I receptor
TβRII: TGF-β type II receptor
α-SMA: α-smooth muscle actin.
